# *GNAS* locus: bone related diseases and mouse models

**DOI:** 10.3389/fendo.2023.1255864

**Published:** 2023-10-18

**Authors:** Wan Yang, Yiyi Zuo, Nuo Zhang, Kangning Wang, Runze Zhang, Ziyi Chen, Qing He

**Affiliations:** ^1^ State Key Laboratory of Oral & Maxillofacial Reconstruction and Regeneration, Key Laboratory of Oral Biomedicine Ministry of Education, Hubei Key Laboratory of Stomatology, School & Hospital of Stomatology, Wuhan University, Wuhan, China; ^2^ School and Hospital of Stomatology, Wuhan University, Wuhan, China

**Keywords:** *GNAS* locus, metabolic bone diseases, mouse models, parathyroid hormone, PTH resistance

## Abstract

*GNAS*is a complex locus characterized by multiple transcripts and an imprinting effect. It orchestrates a variety of physiological processes via numerous signaling pathways. Human diseases associated with the *GNAS* gene encompass fibrous dysplasia (FD), Albright’s Hereditary Osteodystrophy (AHO), parathyroid hormone(PTH) resistance, and Progressive Osseous Heteroplasia (POH), among others. To facilitate the study of the *GNAS* locus and its associated diseases, researchers have developed a range of mouse models. In this review, we will systematically explore the *GNAS* locus, its related signaling pathways, the bone diseases associated with it, and the mouse models pertinent to these bone diseases.

## 1 Introduction

### 1.1 *GNAS* complex locus

**Figure 1:**
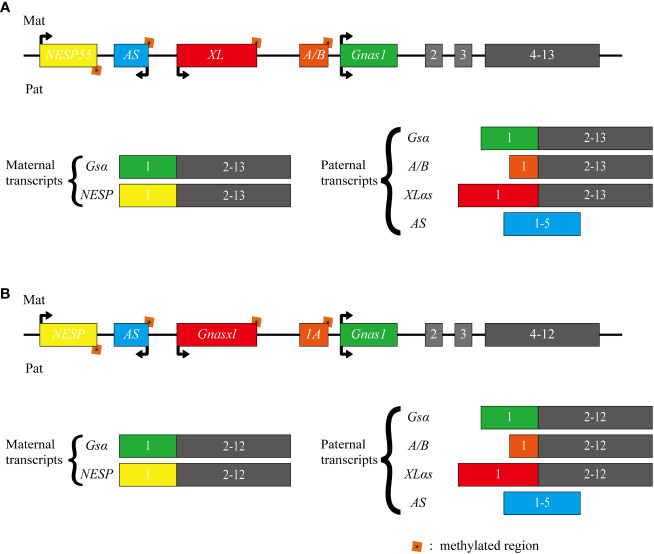
Schematic representation of the human and mouse GNAS locus. The upper section **(A)** illustrates the human GNAS gene locus, while the lower section **(B)** depicts the mouse Gnas gene locus. The GNAS complex locus is characterized by multiple imprinted sense and antisense transcripts. Exons 1-13 of this locus encode Gsα. Differentially methylated promoters lead to several other transcripts, including the maternally expressed NESP55 and the paternally expressed XLαs and A/B (referred to as 1A in mice). Each of these transcripts utilizes distinct first exons that splice onto exons 2-13 of the GNAS gene. Additionally, a non-coding transcript derived from the paternal GNAS allele is produced from the antisense strand (referred to as AS transcript or Nespas in mice). The direction of transcription is indicated by arrows.

The *GNAS* complex locus, located at chromosome 20q13.32 in humans, exhibits a complex and intricate imprinted expression pattern (1). It gives rise to several transcripts, including *Gsα*, *XLas*, *NESP55*, *A/B* (noncoding), and an antisense transcript (*AS*) (**Figure 1**) (2). The *Gsα* transcript contains 13 exons, and several other transcripts originating from *GNAS* locus, such as *A/B, XLas*, and *NESP55*, share exons 2 through 13 with *Gsα*, but utilize their unique promoters and first exons.

In most tissues, *Gsα* is transcribed from both paternal and maternal alleles. However, in specific tissues like renal proximal tubules, thyroid, ovaries, and pituitary, there is a preferential expression of the maternal allele. In contrast, *A/B, XLas*, and *AS* are paternally expressed due to methylation of their maternal promoters at CpG islands within differentially methylated regions (DMRs). While *NESP55* is expressed by the maternal allele, its DMR is methylated on the paternal allele (3–6).

In mice, *Gnas* is located on chromosome 2. While humans have 13 exons in their *Gsα* transcript, mice possess 12 exons in *Gsα* transcript. Nevertheless, both species share the same imprinting and transcription patterns for their transcripts (7, 8).

To date, extensive research has centered on *Gsa*, with limited investigation into other transcripts, especially *XLas*, which is highly similar to *Gsα*. This review aims to provide an updated overview of research on *GNAS*, encompassing related signaling pathways, bone-associated diseases, and mouse models.

Schematic representation of the human and mouse *GNAS* locus. The upper section illustrates the human *GNAS* gene locus, while the lower section depicts the mouse *Gnas* gene locus. The *GNAS* complex locus is characterized by multiple imprinted sense and antisense transcripts. Exons 1-13 of this locus encode *Gsα*. Differentially methylated promoters lead to several other transcripts, including the maternally expressed *NESP55* and the paternally expressed *XLαs* and *A/B* (referred to as *1A* in mice). Each of these transcripts utilizes distinct first exons that splice onto exons 2-13 of the *GNAS* gene. Additionally, a non-coding transcript derived from the paternal *GNAS* allele is produced from the antisense strand (referred to as *AS* transcript or *Nespas* in mice). The direction of transcription is indicated by arrows.

### 1.2 Signaling pathway related to *GNAS*

#### 1.2.1 PTH/PTHrP-Gsα-cAMP-PKA signaling

Protein kinase A (PKA), also known as a cyclic adenosine monophosphate (cAMP)-dependent protein kinase, is a prototypical kinase that exists as a tetrameric holoenzyme in normal conditions (9). It comprises a dimer of regulatory (R) subunits and two catalytic (C) subunits. PKA plays a pivotal role in integrating upstream second messenger signals, such as cAMP, with each step tightly regulated to ensure proper homeostatic signaling. However, mutations in specific genes can disrupt this signaling process, leading to a range of diseases (10).

*Gsα*, the predominant transcript of *Gnas*, primarily participates in the PTH/PTH-related protein (PTHrP)-cAMP-PKA signaling pathway (11). The PTH-activated signal transduction cascade involves G protein-coupled receptors (GPCRs) and numerous downstream intracellular effectors, including heterotrimeric stimulatory G proteins (Gs), cAMP-dependent PKA, and cAMP-specific phosphodiesterases (PDEs). Upon binding of PTH and PTH-related peptide (PTHrP) to the PTH1 receptor (PTH1R), the activation of Gsα stimulates the generation of the intracellular cAMP, thus initiating the signaling pathway (12–14).

In proximal renal tubules, the production of cAMP leads to a decrease in phosphate levels and an increase in vitamin D levels, which is essential for maintaining normal calcium balance. This mechanism could explain the hyperphosphatemia and hypocalcemia observed in patients with pseudohypoparathyroidism caused by inactivating mutations of *Gsα.* Conversely, in patients with paternally inherited pseudopseudohypoparathyroidism (PPHP), their kidneys do not exhibit resistance to PTH, consistent with the preferential expression of *Gsα* maternal alleles in proximal renal tubules, thyroid, ovaries, and pituitary (15). These conditions will be further described in the subsequent sections.

**Figure 2:**
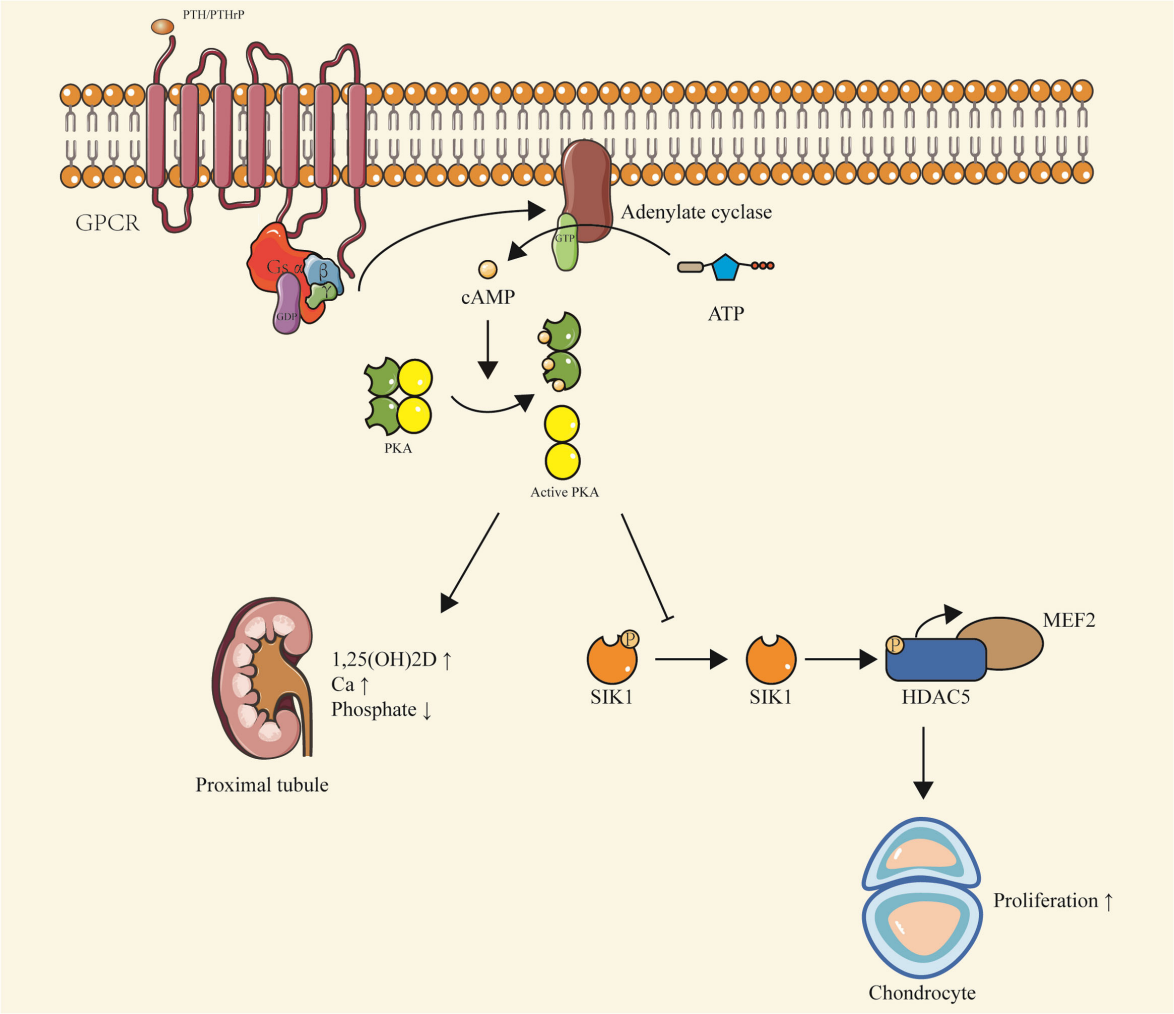
PTH/PTHrP-Gsα-cAMP-PKA signaling. Upon binding of PTH or PTHrP to PTH1R, Gsα is activated, stimulating the production of cAMP. This leads to a series of downstream effects, including the activation of PKA and inhibition of SIK, which regulate MEF2c and promote chondrocyte proliferation. In the proximal renal tubules, cAMP production results in decreased phosphate levels and increased vitamin D levels, contributing to the maintenance of calcium balance. This pathway is implicated in conditions such as pseudohypoparathyroidism and pseudopseudohypoparathyroidism, characterized by hypophosphatemia and hypercalcemia due to inactivating mutations of *Gsα*. The pathway also highlights the preferential expression of *Gsα* maternal alleles in certain tissues, including proximal renal tubules, thyroid, ovaries, and pituitary.

Similar to PTH, PTHrP primarily signals via cAMP. In bone tissues, PTHrP’s interaction with PTH1R is essential for maintaining chondrocyte proliferation during bone development. The production of cAMP activates PKA and inhibits salt-inducible kinase (SIK), subsequently regulating myocyte enhancer factor (MEF2c), ultimately leading to chondrocyte proliferation (16, 17) (**Figure 2**).

#### 1.2.2 Wnt/β-catenin and Hedgehog signaling

Additionally, Gsα can also influence bone development through multiple signaling pathways, such as Wnt/β-catenin and Hh signaling pathways. These pathways are essential for determining the fate of osteoblasts and can also regulate bone mass after bone formation (18, 19). It has been reported that Gsα signaling regulates skeletal development and homeostasis through the Wnt/β-catenin pathway (20). In *Gsα* ^m+/p-^ mice, cortical bone shows elevated expression of the Wnt inhibitors such as sclerostin and Sfrp4, consistent with a decrease in Wnt/β-catenin signaling. Gsα enhances Wnt signaling, and thus promotes the differentiation of mesenchymal progenitor cells into osteoblast lineages (21). Enhanced Wnt-β-catenin signaling pathway has also been observed in bone tissue from FD patients. In these patients, bone marrow mesenchymal stem cells exhibit increased bone progenitor markers and reduced mature osteoblast markers. While activation of Gsα protein alone was not sufficient to activate Wnt/β-catenin pathway, it can enhance Wnt/β-catenin signaling activity by facilitating its association with Lrp5/6 through binding to Axin (22). Thus, Gsα regulates bone formation through at least two distinct mechanisms: promoting mesenchymal progenitor cells to enter the osteoblastic lineage, while enhancing Wnt signaling and inhibiting the differentiation of osteoblasts that have already embarked on the osteoblastic lineage (23).

Furthermore, Gsα signaling acts as an inhibitor of the Hh signaling pathway. Animal studies have demonstrated that the absence of Gsα leads to the upregulation of the Hh signaling pathway. Mechanistically, Gsα inhibits Hh signal transduction by generating cAMP and activating PKA (20, 24).

**Figure 3:**
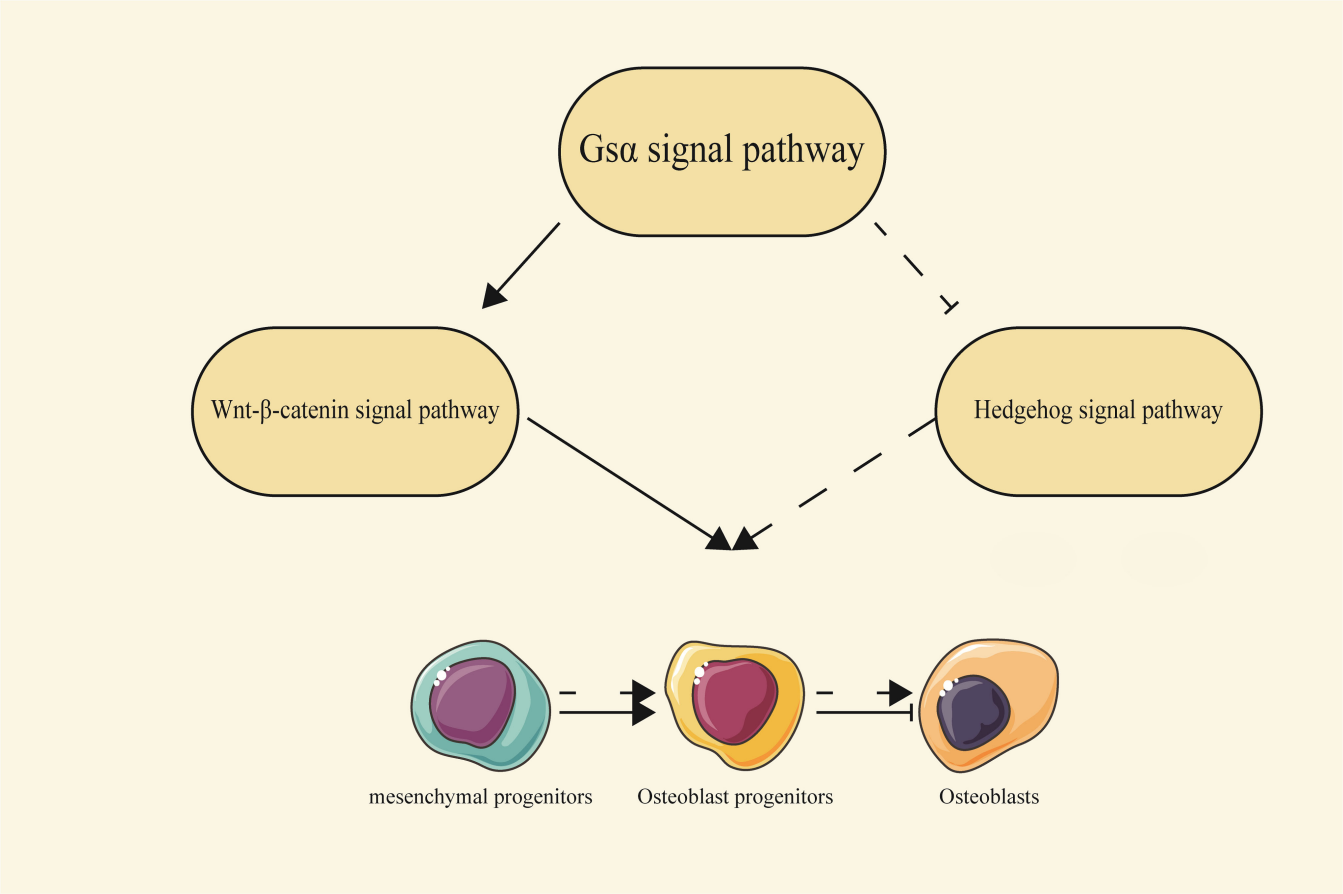
The relationship between the Gsα signaling pathway and the Wnt/β-catenin and Hedgehog signaling pathways. Gsα can regulate the process of osteoblast differentiation by interacting with the Wnt/β-catenin and Hh signaling pathways. Solid arrows represent a promoting effect, solid flat heads represent an inhibitory effect, and dashed lines represent indirect effects.

Both Wnt/β-catenin and Hh signaling pathways act downstream of the Gsα signaling pathway, with Gsα serving as a critical regulator by maintaining a delicate balance between these pathways for proper osteoblast differentiation (**Figure 3**).

#### 1.2.3 XLαs-PKC signaling pathway

At the biochemical level, XLαs shares functional similarities with Gsα by promoting the generation of cAMP upon activation of GPCRs (25, 26). On the other hand, researchers have elucidated the role of XLαs in mediating PTH signaling.

XLαs plays a crucial role in mediating the renal effects of PTH by enhancing Gq/11 signaling, promoting the production of inositol trisphosphate(IP3), and activating specific isoforms of protein kinase C (PKC). This intricate cascade ultimately regulates the homeostasis of phosphate and vitamin D during early postnatal development (27).

Subsequent studies have revealed a novel mechanism involving XLαs/IP3/PKC pathway in the synthesis of Fibroblast Growth Factor 23(FGF23), a hormone originating from bones and bone marrow that play a crucial role in maintaining phosphate balance. FGF23 acts on the kidneys, reducing the reabsorption of phosphate in the proximal tubules and inhibiting the synthesis of 1,25(OH)2D (28).

**Figure 4:**
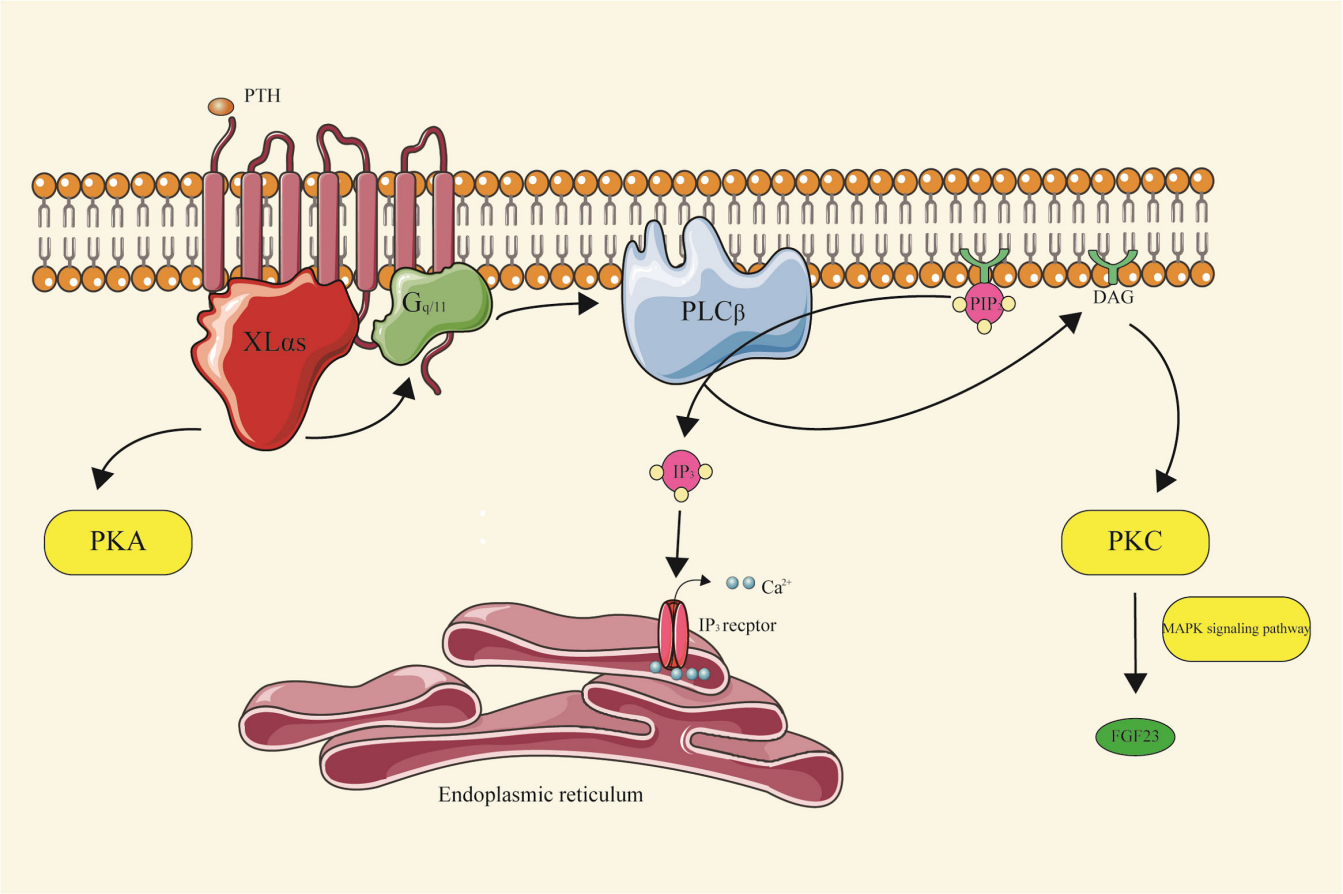
XLαs-PKC signal pathway XLαs, upon activation of GPCRs, promotes the generation of cAMP, a key messenger in cellular signaling. It plays a significant role in mediating the renal effects of PTH. Specifically, XLαs enhances Gq/11 signaling, leading to the production of IP3. This, in turn, activates specific isoforms of PKC, which are crucial in regulating the homeostasis of phosphate and vitamin D during early postnatal development. Furthermore, XLαs is involved in the synthesis of FGF23, a hormone essential for maintaining phosphate balance, through the G protein-coupled IP3/PKC pathway.

The researchers observed that the deletion of XLαs in Ocy454 cells, which resemble osteocytes, resulted in reduced FGF23 mRNA levels, diminished IP3, and decreased PKCα/PKCδ protein levels. In mice, FGF23 production was induced by injecting phorbol myristate acetate (PMA) or by activating Gqα-Q209L in osteocytes and osteoblasts, dependent on the MAPK signaling pathway (**Figure 4**) (29).

Recent advancements have uncovered an intriguing mechanism underlying the activation of phosphoinositide turnover by Gs-coupled receptors. Specifically, investigations have demonstrated that a distinct isoform of phospholipase Cβ (PLCβ), namely PLCβ4, exhibits specific responsiveness to stimulation in cells transfected with XLαs and purified protein components. Notably, PLCβ4 is essential for facilitating the accumulation of phosphatidylinositol in Ocy454 cells following isoproterenol-induced signaling (30).

## 2 Human diseases related to *GNAS*

### 2.1 Human skeletal disorders caused by activating mutations in the *GNAS* gene

#### 2.1.1 Fibrous dysplasia

Fibrous dysplasia (FD) is a rare and complex skeletal disorder characterized by the replacement of bone and marrow by fibrous tissue, resulting in fragile bones (31).The symptoms of FD can involve single or multiple bones, and monostotic fibrous dysplasia is more common than polyostotic, usually involving the femur or craniofacial bones (32–34). In some cases, FD can damage the maxilla or mandible, further affecting the dental development (35, 36). Key clinical features include bone deformities and fractures, which may present various functional impairments, such as vision, hearing, and walking difficulties in severe cases (37, 38).

McCune-Albright syndrome (FD/MAS) is the combination of FD with various extraosseous features. Commonly affected areas include the skin (characterized by hyperpigmented macules known as cafe-au-lait skin spots), a variety of endocrine tissues (resulting in precocious puberty, hyperthyroidism, or growth hormone overload), and occasionally skeletal muscle (intramuscular mucinous tumors, also known as Mazabraud syndrome) (39). FD/MAS exhibits mosaic patterns at the tissue level, with both mutation-positive and mutation-negative osteoblasts present. The presentation varies among patients because of the wide distribution of Gsα and the mosaic nature of FD (40). FD/MAS arises from somatic and functionally acquired mutations in *GNAS*, typically occurring in exon 8 and causes heterozygous missense mutations at R201 position, converting arginine 201 to histidine (R201H) or cysteine (R201C) (41, 42). Occasionally, arginine can also be replaced by serine, glycine, or leucine (43). Different codons may also be affected, for instance, codon 227 (in exon 9; Q227), which can lead to FD (44). Mouse and *in vitro* models based on these mutations disrupt Gsα’s intrinsic GTPase activity during skeletal development, leading to constitutive activation and excessive cAMP production (45). Constitutive Gsα signaling impairs the differentiation of mutant osteoprogenitor cells, as observed in *in vitro* studies of cells isolated from FD lesions and normal human osteoprogenitor cells stably transduced with *Gsα* ^R201C^ (23, 46). Proliferation of undifferentiated skeletal stem cells produces fibrous bone tissue, accompanied by the loss of normal bone marrow functions like hematopoiesis. Thus, FD is characterized by a lack of hematopoietic cells and adipocytes in the bone marrow, which is replace by fibrotic tissue. Abnormal bone structure, mineral content, and trabecular manifestations stem are attributed to aberrant osteoblast activity, potentially through Wnt/β-catenin signaling. Osteoclast production may also be promoted locally by osteoclast-promoting factors, such as interleukin-6 and receptor activator of nuclear kappa-B ligand. Additionally, inherent mineralization defects and phosphorylated hormone fibroblast growth factor-23 (FGF23) produced by FD injury contribute to severe osteomalacic changes (20–23, 47).

### 2.2 Human skeletal disorders caused by inactivating mutations in the *GNAS* gene

#### 2.2.1 Albright’s hereditary osteodystrophy

Albright’s Hereditary Osteodystrophy (AHO) is a condition caused by mutations in the *GNAS* gene, characterized by various physical traits, including short stature, brachydactyly, obesity, a round face, cognitive impairment, and the abnormal formation of bone outside the skeletal system, referred to as heterotopic ossification (48, 49). These features were first described by Albright et al., hence the name Albright’s hereditary osteodystrophy (50, 51).

Heterotopic ossification is the most devastating feature of AHO. It involves the formation of bone in soft tissues, primarily through intramembranous ossification (52–54). Ectopic ossification in patients with AHO occur within the dermis and subcutaneous tissue, causing painful lesions that may require surgical intervention. These lesions can appear spontaneously or be triggered by various factors from minor trauma to major surgery or extensive wounds (55). Histological analysis shows the presence of mineralized bone tissue with a bone marrow component in the biopsy specimen, while no cartilage is detected, indicating an origin primarily in intramembranous bone formation. Importantly, these ectopic ossifications can occur regardless of whether the *Gsα* mutation is inherited from the mother or father, reflecting Gsα haploinsufficiency. Notably, they are not associated with abnormal levels of calcium and phosphate (56, 57). Gsα plays a vital role in ectopic ossification, since reduced Gsα levels in human mesenchymal stem cells lead to increased bone formation and inhibited adipocyte formation (20, 58). Removing Gsα from progenitor cells expressing osterix reduces the number of bone progenitor cells while enhancing osteoblast differentiation. Additionally, Runx2, a gene marker for osteoblasts, seems to inhibit the expression of Gsα, suggesting an opposing role of Gsα in osteogenic differentiation (59).

#### 2.2.2 Pseudohypoparathyroidism

Pseudohypoparathyroidism (PHP) constitutes a rare group of endocrine disorders, initially reported by Albright and colleagues in 1942, characterized by complete or partial failure of end-organ to respond to PTH. PHP is manifested by variable degrees of hypocalcemia and hyperphosphatemia (51). The exact global prevalence of PHP is uncertain, but is estimated to be around 0.79 cases per 100,000 people, with a slightly higher prevalence of 1.2 cases per 100,000 in Japan (60, 61).

PHP is classified according to the level of urinary cAMP after bovine parathyroid extract injection. PHP type I (PHP I) differs from PHP type II (PHP II) by the abnormal urinary cAMP response to exogenous PTH stimulation. Within PHP I, there are three subtypes (Ia, Ib, and Ic), depending on the specific defects in the *GNAS* gene. These defects can result from heterozygous inactivating mutations or imprinting defects on the maternal allele. When heterozygous inactivating mutations are in the paternal allele, the mutations lead to pseudo-pseudohypoparathyroidism (PPHP) and progressive osseous heteroplasia (POH). PHP Ia/PHP Ic and PHP Ib are distinguished by the presence or absence of AHO. Hormonal resistance helps distinguish PHP I from PPHP. Additionally, an *in vitro* assay that measures Gsa protein activity from erythrocyte membranes can differentiate between PHP Ia and PHP Ic, as Gsα activity is reduced in PHP Ia but normal in PHP Ic, although this may be attributed to the assay conditions rather than true biological differences (61, 62). These PHP subtypes are typically caused by genetic mutations inherited in an autosomal dominant manner, or by epigenetic, sporadic, or genetic changes within or upstream of the *GNAS* gene (**Table 1**).

**Table 1:** Genetics and clinical characteristics of inactivating *GNAS* mutation diseases.

	PHP Ia	PHP Ib	PHP Ic	PHP II	PPHP	POH
Inheritance characteristic	AD	AD/Sporadic	AD	Sporadic	AD	AD/Sporadic
Parental origin	Maternal	Maternal	Maternal	Unclear	Paternal	Paternal
Genetic defects	1) Heterozygous structural mutations in the *GNAS* gene 2) Mutation hotspot in exon 7 (c.568_571delGACT) 3) Heterozygous c.715A>G (p.N239D) mutation in exon 9 4) Two mutation hotspots in exon 6 (p.R166C) and exon 5 acceptor splice site (c.435 + 1G>A)	**Familial PHP Ib** 1) Abnormal methylation at the *GNAS A/B*, *AS1*, *NESP55*, and *XLαs* DMRs 2) 3-kb deletion of exon *A/B* locating in the *STX16* gene 3) 4.2-kb microdeletion in the *NESP55* exon 4) 18,988-bp deletion removes *NESP55* and most of *GNAS AS* intron 4 **Sporadic PHP Ib** 1) Methylation defects of patUPD20q including *GNAS*	1) Heterozygous mutations in exon 13 of the *GNAS* gene (p.E392K, p.E392X, p.L388R)	Unclear	1) Heterozygous mutations in the *GNAS* gene	1) Heterozygous mutations in the *GNAS* exons 2-13, and less frequently exon 1 2) (c.175C > T, p.Q59X) in exon 2
Endocrine defects	Resistance to multiple hormones, such as PTH, TSH, GHRH, LH/FSH, etc.	Resistance limited to PTH and TSH	Resistance to PTH, TSH	Resistance to PTH	No	No
Serum PTH level	Increased	Increased	Increased	Increased	Normal	Normal
Serum calcium level	Decreased	Decreased	Decreased	Decreased	Normal	Normal
Serum phosphate levels	Increased	Increased	Increased	Increased	Normal	Normal
Obesity	Often early-onset obesity	No obesity or early-onset obesity	Often early-onset obesity	No	No	No/Often slim
Short stature	Yes	No	Yes	No	Yes	Not enough evidence
Brachydactyly	Yes	No/Mild	Yes	No	Yes	Infrequent
Prenatal growth retardation	Slightly low birth weight	normal	Slightly low birth weight	normal	Small for gestational age	Small for gestational age
Heterotopic ossifications	Superficial subcutaneous ossification	No	Superficial subcutaneous ossification	No	Superficial subcutaneous ossification	Heterotopic ossification extending into deep connective tissue and muscle

PHP Ia, pseudohypoparathyroidism type Ia; PHP Ib, pseudohypoparathyroidism type Ib; PHP Ic, pseudohypoparathyroidism type Ic; PHP II, pseudohypoparathyroidism type II; PPHP, pseudo-pseudohypoparathyroidism; POH, progressive osseous heteroplasia; AD, autosomal dominant inheritance; DMR, differentially methylated region; PTH, Parathyroid hormone; TSH, thyroid-stimulating hormone; GHRH, growth hormone-releasing hormone; LH/FSH, luteinizing hormone and/or follicle-stimulating hormone.

The previous classification of PHP did not consider the molecular defects, omitting conditions like acrodysostosis, POH, and PTH1R-related chondrodysplasia (63). A new classification system has been established by the EuroPHP network, encompassing all disorders related to inactivation of the PTH/PTH1R signaling pathway (iPPSD). This nomenclature employs numerical identifiers to characterize specific subtypes, enabling the description of both clinical and molecular features. For instance, iPPSD1 represents a loss-of-function variant in PTHR1, iPPSD2 corresponds to a loss-of-function alteration in *GNAS*, iPPSD3 denotes methylation defects at one or more *GNAS* DMRs, iPPSD4 signifies a pathogenic variant in PRKAR1A, iPPSD5 indicates a pathogenic variant in PDE4D, iPPSD6 relates to a pathogenic variant in PDE3A, and iPPSDx is used when no molecular defect is identified. These classifications focus on categorizing conditions based on their underlying mechanisms and can aid in guiding diagnosis through major and minor diagnostic criteria (64, 65).

##### 2.2.2.1 PHP Ia

Patients with PHP Ia usually have typical AHO manifestations (66, 67). The clinical presentation is notably intricate due to the multifaceted effects of *GNAS* coding mutations. These mutations can lead to hormone resistance against PTH, thyroid-stimulating hormone (TSH), luteinizing hormone (LH), follicle-stimulating hormone (FSH), and growth hormone-releasing hormone (GHRH). The extent of hormone resistance may vary among affected tissues, contributing to the complexity of the phenotype observed in PHP1a patients. Clinical features are influenced by whether *Gsα* transcription is mono- or biallelic in various tissues. In PHP1A, the most encountered hormonal resistance is PTH resistance in the renal proximal tubule. However, due to biallelic expression of *Gsα* in the distal tubule, urinary calcium reabsorption remains relatively normal in patients with PHP1A. Moreover, the specific phenotype also depends on the stage of development. Notably, the development of hypocalcemia due to PTH resistance in the renal proximal tubule typically occurs after infancy. It is noteworthy that TSH levels in PHP1A can be elevated at birth, followed by a period of normalization for 9–20 months before increasing once more (68, 69). In some instances, specific *GNAS* mutations in PHP1a patients can lead to a condition known as testotoxicosis. This condition involves excessive testicular stimulation by LH and FSH, resulting in precocious puberty and virilization in males (70, 71).

PHP Ia subtype is identified by sluggish urinary cAMP and phosphate excretion in response to exogenous PTH administration (72). Since PTH inhibits phosphate reabsorption and induces 25-dihydroxy vitamin D 1-α-hydroxylase (Cyp27b1) mRNA expression through the cAMP-dependent cellular mechanism, patients may develop not only hyperphosphatemia but also hypocalcemia. Serum PTH levels remain elevated in these patients (73). Notably, obesity and cognitive impairment occur predominantly in patients with PHP-Ia due to maternal transmission of the *Gsα* mutation (74, 75).

The genetic basis of PHP Ia involves heterozygous inactivating mutations affecting maternal *GNAS* exons 1-13. The most common mutation types are frameshift and missense mutations, accounting for 77% of cases. Other mutation types include nonsense, splice site mutations, in-frame deletions or insertions, and gene deletion mutations (76, 77). Over 180 different mutations in *GNAS* have been identified in PHP-Ia, with two-thirds being unique. These mutations are distributed throughout the *GNAS* coding region. However, deletion mutations in the four nucleotides of GACT at sites -568 to -571 in the coding region of exon 7 (p.D190MfsX14) have been frequently reported. Besides, heterozygous c.715A>G (p.N239D) mutation was identified in exon 9 of the *GNAS* gene (2, 67, 78). Susanne Thiele et al. detected two novel hotspots, one in exon 6 (p.R166C) and another in the exon 5 acceptor splice site (c.435 + 1G>A) (79).

##### 2.2.2.2 PHP Ib

PHP Ib is primarily familial and is associated with imprinting defects upstream of the maternal *GNAS* gene (80). Most patients with PHP Ib show resistance limited to PTH and TSH but may exhibit occasional signs of AHO. Imprinting of the *GNAS* locus results in the absence of maternal *Gsα* expression in renal tissue (81).

Familial PHP Ib is driven by epigenetic aberrations within the DMR of *GNAS* that can affect multiple transcripts, including *Gsα, XLαs*, *NESP55*, *A/B*, and *AS*. Studies have shown that small deletions of 3-kb in the *STX16* gene fragment and 4.2-kb microdeletion in the *NESP55* exon can cause methylation defects in maternal exons *A/B*, which are also involved in PHPIb pathogenesis (82–85). Loss of methylation at exon *A/B* and the resulting biallelic expression of *A/B* transcripts reduces *Gsα* expression, leading to hormone resistance. Furthermore, Richard et al. identify a deletion of 18,988 bp that removes *NESP55* and a large part of its counterpart *GNAS AS* intron 4 in a PHP Ib family (86). A recent study has revealed that imprinting defects in familial PHP Ib can emerge during the postzygotic period. The active long-range interaction between STX16-putative imprinting control region (ICR) and NESP-ICR plays a critical role in A/B DMR methylation, as demonstrated through the generation of a human embryonic stem cell model for this condition. Consequently, correcting A/B DMR hypomethylation during this timeframe holds promise as an effective treatment approach for PHP Ib patients (87). Although the extensive or partial loss of imprinting affecting all *GNAS* DMRs is often detected in PHP Ib cases, it has been associated with comprehensive loss of methylation of the maternal *GNAS* gene and paternal uniparental isodisomy or heterodisomy of chromosome 20q (patUPD20q) in the sporadic PHP Ib (88).

##### 2.2.2.3 PHP Ic

PHP Ic is a variant of PHP Ia that combines AHO and hormonal resistance with normal Gsα activity *in vitro*, which is induced by receptor coupling defects in the common cAMP signaling pathway as PHP Ia. Different molecular defects of pathogenic mutations in the *Gsα* encoding exons of the *GNAS* gene have been identified in patients with PHP Ic (89, 90).

PHP Ic also inherits through autosomal dominant inheritance and was originally described to be associated with a mutation in exon 13 of the maternal *GNAS* gene, which can affect the C-terminus of *Gsa* and cause receptor coupling dysfunction (91). In addition, Susanne Thiele et al. have further identified three different heterozygous mutations (p.E392K, p.E392X, p.L388R) in exon 13 that affect the two residues 388 and 392 in the carboxy-terminal portion of *Gsα* (89).

##### 2.2.2.4 PHP II

First described by Drezner et al. in 1973, PHP II is a clinically rare and often sporadic form of PHP (90). Patients with PHP II have normal nephrogenic excretion of cAMP with impaired phosphatic urinary phosphate excretion due to exogenous PTH administration. Until now, PHP II cases are infrequently documented (92, 93). In contrast to PHP I, the underlying mechanisms of abnormal PTH signaling in PHP II are still elusive. Nevertheless, there is a prevailing suspicion that PHP II may result from an acquired deficiency secondary to vitamin D insufficiency or a malfunction in signaling pathways downstream of Gsα. This parallels the aberrant signaling observed in patients with acrodysostosis attributed to mutations in the PRKAR1A or PDE4D genes, both of which are downstream to cAMP generation (94–96). In addition, Yamada et al. studied the effects of serum immunoglobulin G from a patient with PHP-II and Sjögren’s syndrome on renal function in rats (93). The study showed that autoantibodies react with components of the renal tubular plasma membrane and can block the progression of PTH-induced phosphaturia (92). The underlying molecular defect remains unknown due to the small number of reported PHP II cases.

#### 2.2.3 PPHP

Patients affected by PPHP show physical characteristics of AHO but do not experience hormone resistance (97). It is characterized by normal PTH, calcium, and phosphorus levels (98). The pathogenesis involves a heterozygous loss-of-function mutation in the paternal *GNAS* gene, resulting in decreased Gsa activity *in vitro*. PPHP Patients may exhibit short stature, brachydactyly but generally do not have mental retardation or obesity. These patients tend to be smaller by gestational age and have lower birth weights compared to those with PHP Ia, and patients carrying exon 2-13 mutations were even smaller at birth than those carrying exon 1 mutations, suggesting that the paternally expressed *GNAS* proteins, including XLαs is involved in fetal growth (99, 100).

#### 2.2.4 POH

POH represents another disorder in which patients often develop cutaneous ossifications early in life, involving deep connective tissue and skeletal muscle (101). Ectopic ossification of POH is formed mainly through intramembranous osteogenesis. However, a histopathological examination of subcutaneous ossification in one patient revealed the presence of chondrocyte clusters, a feature found in fibrous developmental abnormalities. Most lesions in patients begin in infancy with cutaneous or subcutaneous ossification and subsequently become invasive, and patients often present with severe ankylosis of the affected joints, which limits growth. In most patients with POH, heterotopic bone formation appears to be an isolated clinical manifestation with no other typical AHO features, while most *GNAS* mutations causing POH are paternally inherited (102–104).

Mutations in paternal *GNAS* exons 2-13, and less frequently exon 1, cause this severe form. This also leads to the hypothesis that POH is primarily related to a deficiency of the paternal XLαs protein rather than a deficiency of the paternal Gsα. Recently, Jing Ma et al. identified a *de novo* mutation (c.175C > T, p.Q59X) in exon 2 of the *GNAS* gene in a clinical case of POH (105–107).

## 3 *GNAS*-related mouse models

Various of *Gnas* gene-associated mouse models have been developed, serving as invaluable tools to deepen our comprehension of the *Gnas* locus, and ultimately promote the advancement of targeted therapeutic strategies for affected individuals. In the following sections, we will discuss these model mice with respect to different transcripts originated from *Gnas* (**Table 2**).

**Table 2:** *Gnas* knockout mice and phenotypes.

Model	Influenced genes	Phenotype
MatDp(dist2)	Maternal transcripts	Long, thin body, inability to suckle, lack of vigor, high postnatal mortality
PatDp(dist2)	Paternal transcripts	Short and square body, hyperactivity, hypoglycemia
*Oed-Sml*(maternal)	Maternal transcripts	Perinatal edema, pre-weaning lethality (genetic background associated), increased BAT, tiny heart, subcutaneous ectopic ossification
*Oed-Sml*(paternal)	Paternal transcripts	High prenatal mortality, reduced BAT, postnatal growth retardation, adult wasting, and subcutaneous heterotopic ossification
*Gnas* exon2^m+/p-^	Paternal transcripts	Decreased suckling, 77% perinatal mortality, decreased adipose tissue, decreased activity, postnatal growth retardation, and narrow body, decreased survivor fat content, increased metabolic rate, increased activity levels, glucose tolerance, insulin sensitivity, and lipid clearance, fibroids on ears, paws, and tail at four months of age, exhibiting calcification
*Gnas* exon2^m-/p+^	Maternal transcripts	perinatal edema, 80% pre-weaning lethality, increased fat content, four-square bodies, tremors, and imbalances, surviving mice becoming obese, slowed metabolism and reduced activity, fibroids on ears, paws, and tail at four months of age, exhibiting calcification
*Gnas* exon1^m+/p-^	Paternal *Gsα*	Subcutaneous ectopic ossification, impaired bone formation, and increased bone resorption resulted in decreased bone parameters in mice, skulls appeared to have a more rounded and dome-like structure, and the length of the spheno-occipital synchondrosis (SOS) was significantly reduced
*Gnas* exon1^m-/p+^	Maternal *Gsα*	Hormone resistance, subcutaneous osteomalacia, enhanced bone parameters due to increased osteoblast activity and normal bone resorption, cranial osteomalacia, and enhanced cranial bone formation, skulls appeared to have a more rounded and dome-like structure, and the length of the spheno-occipital synchondrosis (SOS) was significantly reduced.
*Gnasxl* ^m+/p-^	*XLαs*	Stenosis, lean body mass, reduced suckling, weakness and inactivity, growth retardation, high postpartum mortality, decreased blood glucose levels, low pancreatic hyperglycemic concentrations, increased glucose tolerance and insulin sensitivity, reduced adipose tissue, hyperphosphatemia, hypocalcemia, increased serum concentrations of PTH and 1,25-dihydroxy vitamin D, decreased abundance of the sodium-phosphate cotransporter Npt2a in renal brush membranes, and slightly increased PTH-induced urinary excretion of cAMP
*NESP55* KO DeltaNesp55(p) DeltaNesp55(m) ΔNesp55^m^	*NESP55* Paternal NESP55DMR Maternal NESP55DMR	Normal viability and metabolism Normal Hypocalcemia, hyperphosphatemia, and secondary hyperparathyroidism, early postnatal lethality

### 3.1 Chromosome 2 distal duplication mouse model

In early studies on chromosomal variability in mice, Cattanach and Kirk found mice with two maternal duplications of the distal region of chromosome 2 (MatDp(dist2)) that have long, thin bodies that cannot suck and die within a few hours after birth. In contrast, mice with paternal duplications of this region (PatDp(dist2)) have the opposite phenotype: short, square bodies, hyperactive, and death within a few days after birth (108). Subsequent research on PatDp(dist2) has demonstrated that the majority of its phenotypes result from the combined effects of *XLαs* overexpression and *Gsα* expression deficiency. This suggests that *XLαs* and *Gsα* may exhibit antagonistic roles in some tissues, contributing to a wide range of phenotypic effects. Furthermore, it has been concluded that the monoallelic expression of *XLαs* and *Gsα* is essential for normal postnatal growth and development (3, 109, 110).

### 3.2 Oed-Sml mouse model

Similarly, a point mutation in exon 6 of *Gnas* (*Oed-Sml* point mutation) causes abnormalities in *Gsα* and *XLαs*. Mutations of paternal origin result in Sml phenotype with substantial preweaning loss, decreased brown adipose tissue (BAT), hypoglycemia, postnatal growth retardation, and leanness in adulthood, whereas mutations of maternal origin cause Oed phenotype with perinatal edema, pre-weaning associated with genetic background lethality, increased BAT, and micro cardia (111, 112) The metabolic phenotypes of Oed and Sml mice were determined by Kelly et al., who found that adult Oed and Sml mice had opposite metabolic phenotypes when fed a high-fat diet. In the maternal line, the obese Oed phenotype was attributable to non-functional full-length *Gsα*. In contrast, in the paternal line, Sml mice were smaller and resisted the development of obesity on a high-fat diet, and these effects were attributable to mutant *XLαs* (113). Cheeseman et al. reported subcutaneous ossification and benign cutaneous fibroepithelial polyps in this model, phenotypes that can occur in both the maternal and paternal line of inheritance of missense mutations and are therefore caused by Gsα (114). It is worth mentioning that maternally inherited *Oed-Sml* mice lacking the paternal exon *1A* exhibit attenuated PTH resistance and derepression of *Gsα* expression in BAT. These findings are consistent with the silencing function of the murine *1A* transcript, which reduces the bioactivity of Gsα (115).

### 3.3 *Gnas* exon2 knockout mouse model

Yu et al. generated the initial *Gnas* knockout mice (*Gnas* exon2^m+/p-^, *Gnas* exon2^m-/p+^) by disrupting the second exon of the *Gnas* gene (116). In subsequent studies, they discovered that neonatal mice with paternal deficiency displayed reduced suckling, 77% perinatal mortality, diminished adipose tissue (BAT and White adipose tissue (WAT)), inactivity, postnatal growth retardation, and a narrower body. Conversely, newborn mice with maternal deletion presented perinatal edema, 80% preweaning lethality, increased adiposity (BAT and WAT), square-shaped bodies, tremors, and imbalance. Surviving E2^m-/p+^ mice became obese, hypometabolic, and hypoactive, whereas E2^m+/p-^ survivors had reduced adiposity, with increased metabolic rate, activity levels, glucose tolerance, insulin sensitivity, and lipid clearance (116–118). We now understand that this mutation, when maternally inherited, effectively eliminates *Gsα* expression in imprinted tissues and results in a 50% reduction in tissues with biallelic expression. It may also interfere with the *Nesp* transcript. On the other hand, when the mutation is paternally inherited, it affects transcripts *XLαs*, *Gsα*, and the noncoding exon *1A*. The phenotype of heterozygotes carrying maternally inherited exon2 mutations (*Gnas* exon2^m-/p+^) has later been confirmed to be equivalent to that of heterozygotes with *Gnas* exon1 mutations specifically affecting Gsα (*Gnas* exon1^m-/p+^) (119, 120).

Subsequent research on this model revealed that mice with maternal or paternal allelic disruption of the second exon of the *Gnas* gene developed fibromas or angiofibroma on their ears, claws, and tails, beginning at four months of age. These tumors exhibited calcification, which appeared to be secondary calcification of fibrotic lesions associated with an abundance of matrix metalloproteinase 2, and the absence of osteoblast-specific markers indicated that the calcification was related to extracellular matrix degradation and not secondary to ossification. These findings suggest that *Gnas*E2^m-/p+^ mice may serve as a useful model for further investigation of the mechanisms underlying skin fibroblast proliferation and calcification (121).

All of the mouse models mentioned above disrupt several transcripts simultaneously, and we will further compare the mutant mouse models with distinct transcript modifications next.

### 3.4 *Gsα* exon1 mouse model

Germain-Lee et al. generated *Gsα*-specific knockout mice (CD-1 background) by specifically targeting the disruption of the first exon of the *Gnas* gene in order to investigate the effects of an isolated deficiency of *Gsα.* The maternal inheritance of this mutation successfully recapitulated the key features of human PHP1a, including hormone resistance. Notably, significant differences were observed between these mice and those with previously reported exon 2 disruptions. This finding indicates that the loss of *Nesp55* and/or *XLαs* may contribute to the abnormalities observed in mice with exon 2 disruptions (120, 122).

In a similar fashion, Chen et al. generated a mouse model by disrupting the first exon of *Gsα*, resulting in the ablation of Gsα. Homozygous mutations proved to be embryonically lethal, which is in line with findings in mice with exon 2 disruptions. Interestingly, the paternally inherited heterozygous mutants (E1^m+/p-^) demonstrated normal survival rates while displaying obesity and insulin resistance, which contrasts with the phenotypes observed in E2^m+/p-^ and *XLαs* knockout mice. Alternative *Gnas* gene transcripts seem to have opposing effects on glucose and lipid metabolism. Additionally, the maternally inherited mutant mice (E1^m-/p+^) exhibited more severe obesity and insulin resistance than their paternally inherited counterparts, along with lower metabolic rates. Notably, the metabolic differences between E1^m-/p+^ and E1^m+/p-^ mice were not attributed to hypothyroidism, as there were no significant differences in serum TSH and free thyroxine (T4) levels between mutant mice and their wild-type counterparts (119).

Huso et al. initially reported subcutaneous osteophytes (SCO) in *Gnas* E1^m-/p+^ mice, demonstrating progressive development of SCO in these animals over time. The number and size of ossified lesions increase, and they are primarily located in the dermis, often in perifollicular regions and subcutaneously. Interestingly, these lesions are particularly prominent in skin areas susceptible to injury or stress. Notably, ossification can be uniformly detected in adult mice before one year of age, and it is more extensive in male mice than female mice. These animal models offer a valuable system for investigating the pathogenesis of SCO formation and developing novel therapies to target ectopic bone formation (123).

On the other hand, Pignolo et al. used an F1 mouse model of SvEv × CD1 background with E1 mutation (120, 122) to determine that this mutation(E1^m+/p-^) upregulated multiple transcripts occurring in the osteogenic differentiation of wild-type adipose stromal cells (*Gsα, XLαs, NESP, 1A*), while simultaneously accelerating adipose stromal cell osteogenic differentiation. *In vivo*, the osteoblast alterations in E1^m+/p-^ manifest as subcutaneous ectopic ossification, which can be detected as early as nine months of age. These findings suggest that Gsα is a regulatory factor in determining the fate of adipose-derived mesenchymal progenitor cells, particularly in terms of their involvement in bone formation (124).

Liu et al. further investigated the effects of paternally inherited heterozygous inactivation of *Gsα* (E1 ^m+/p-^)on adipose tissue. They found that it impairs the lipogenic differentiation of adipose stromal cells and reduces the expression of CCAAT-enhancer binding protein (C/EBP) β, C/EBPα, peroxisome proliferator-activated receptor γ (PPAR-γ), and adipocyte protein 2. Not only that, this damage could be rescued by an adenylate cyclase activator-forskolin and provided evidence that Gsα-cAMP signaling is necessary in the early stages of the process. *In vivo*, *Gsα*E1 ^m+/p-^ mice showed a significant decrease in both adipose tissue weight and lipogenesis-related marker genes, and this inhibition of adipose tissue enhanced the expression of some osteogenic marker genes. Their study supports the hypothesis that Gsα regulates soft tissue bone lipid homeostasis (125).

Ramaswamy investigated the effects of *Gsα* heterozygous inactivation on bone modeling and remodeling and for early bone development and modeling, and found that both *Gnas* ^m+/p-^ and *Gnas* ^m-/p+^ mice had lower body weight and femur length compared to WT, with the paternal mutation showing a greater reduction. The femur of both mutants showed a significant reduction in stiffness and peak load consistent with the μCT data. For bone remodeling, the authors analyzed 3- and 9-month-old mice; paternally inherited *Gsα* ^m+/p-^ mice had reduced body weight and shorter femurs, while Gsα^m-/p+^ had increased body weight and no difference in femur length from wild-type controls; uCT analysis of the cortical bone of the mid-femoral diaphysis showed that *Gsα* ^m+/p-^ mice at months 3 and 9 had significantly lower total cortical bone volume, cortical thickness, and In contrast, maternally mutant mice showed no difference in cortical bone volume and thickness compared to WT; the femur was weaker in both age groups of *Gsα* ^m+/p-^ mice by the three-point bending test, with significantly lower peak load and stiffness compared to WT; bone strength was unaffected in either age group of *Gsα* ^m-/p+^ mice. Not only that, the decrease in bone mass in *Gsα* ^m+/p-^ was not due to an effect on osteoblasts but rather due to an increase in the number of osteoclasts resulting in endosteal resorption. The authors further showed by flow cytometry that the cortical bone defect in *Gsα* ^m+/p-^ was not caused by an increase in osteoclast precursors in the bone marrow. *In vitro* osteoclast differentiation experiments showed that. A reduction in Gsα signaling enhanced osteoclast differentiation and osteoclast resorption activity, and cells deleted from the paternal *Gnas* allele had a greater effect than the maternal allele and were accompanied by a reduction in pCREB, β-catenin and cyclin D1 and enhanced Nfatc1 levels during differentiation. Forskolin treatment that elevate adenylyl cyclase and PKA activity increased pCREB, decreased Nfatc1, and rescued osteoclast differentiation in *Gsα* ^m+/p-^, while cortical bone in *Gsα* ^m+/p-^ mice showed sclerostin and elevated Sfrp4 (Wnt inhibitor) were elevated. These suggest a novel role for Gsα in maintaining bone mass through the cAMP/PKA and Wnt/β-catenin pathways regulating osteoclast differentiation and function (21).

Patrick McMullan et al. investigated the differences in bone remodeling between paternally inherited and maternally inherited *Gnas* heterozygous inactivation mutations, particularly in female mice. These differences were observed mainly in *Gnas*E1 ^m+/p-^ mice, which showed decreased bone parameters due to impaired bone formation and increased bone resorption. However, *Gnas*E1 ^m-/p+^ mice exhibited enhanced bone parameters due to increased osteoblast activity and normal bone resorption. This increased osteoblast activity may be secondary to the partial resistance of osteoclast lineage cells to calcitonin. This provides the first direct evidence that Gsα affects osteoblast-osteoclast interactions and differential effects on bone remodeling based on different mutation inheritance patterns (126).

Neetu Krishnan et al. studied the cranial phenotype of the AHO mouse model, and they found that the skulls of *Gnas* E1^m-/p+^ and *Gnas* E1^m+/p-^ mice appeared to have a more rounded and dome-like structure compared to wild-type mice. The cranial lengths of *Gnas* E1^m-/p+^ and *Gnas* E1^m+/p-^ mice were significantly shorter at 3 and 12 weeks of age compared to wild-type were significantly shorter compared to wild-type. Compared to WT mice, *Gnas* E1^m-/p+^ mice exhibited a mild but statistically significant increase in cranial height at 12 weeks of age. As well, *Gnas* E1^m-/p+^ mice showed enhanced cranial osteogenesis as well as cranial bone formation. At P7, the length of spheno-occipital synchondrosis (SOS) was significantly reduced in *Gnas* E1^m-/p+^ and *Gnas* E1^m+/p-^ mice compared to wild-type. The craniofacial abnormalities observed in *Gnas* E1^m+/p-^ mice may be related to abnormal skeletal patterns secondary to premature SOS closure due to accelerated proliferation of chondrocyte differentiation (127).

### 3.5 *Gsα* conditional knockout mouse model

Researchers have employed various Cre Recombinase mice to investigate the role of Gsα in specific tissues and cells. In this section, we delve into the findings of Gsα conditional knockout models, particularly those with bone lineage-specific inactivation (**Table 3**).

**Table 3:** Phenotypes of bone-associated *Gsα* conditional knockout mice.

Cre-line used	Specific cell	Phenotype
*Col1α*-cre	Osteocyte/Osteoblast	Craniofacial skeletal dysplasia; defective primary spongiosa formation in long bones, reduced immature osteoid (new bone formation) and the total length, reduced volume of bone trabeculae, thickened cortical bone
*Prx1*-cre	Mesenchymal cell	Born with interphalangeal webbing, joint fusion, and progressive heterotopic ossification in the soft tissues, most mutant mice die from extensive bone and joint fusion and tendon mineralization; accelerated cranial bone formation during cranial development and cranial deformities after birth, Craniosynostosis.
*Osterix*-cre	Osteoblast	Severe osteoporosis, fractures at birth, did not survive to weaning
*Dmp1*-cre	Osteocyte	Bone marrow aplasia, low bone mass, reduced adipose tissue, gender variability

#### 3.5.1 Osteoblast/Osteocyte

Akio Sakamoto et al. used *Gsα*-floxed mice (128) with collagen Iα1 promoter Cre recombinase transgenic mice (129) to create osteoblast/osteocyte-specific Gsα deficient (BGsKO) mice. These mice exhibited similarities to PTH deficient mice (130). In neonatal BGsKO mice, craniofacial skeletal dysplasia was observed, with thickening of the mandible, maxilla, and zygomatic arch, especially Merkel’s cartilage, which had ossified in neonatal BGsKO mice. Long bones in BGsKO mice displayed defective primary spongiosa formation, immature osteoid (new bone formation), and reduced total length, leading to decreased trabecular volume. In contrast, cortical bone thickened with a narrowed bone marrow cavity, possibly due to reduced cortical bone resorption caused by a decrease in osteoclasts on the cortical bone surface. Altered expression of alkaline phosphatase and reduced expression of bone-bridging protein and osteocalcin suggested a decrease in mature osteoblasts in the bone. The expression of osteoclast-stimulating factor receptor activator of NF-κB ligand (RANKL) was also reduced (131).

Zhang et al. investigated transgenic mice with osteoblastic *Gsα* overexpression (HOM-Gs mice) driven by the 3.6-kilobase (kb) *Col1A1* promoter. Both male and female HOM-Gs mice displayed elevated bone turnover, overactive osteoblasts, and osteoclasts, culminating in a high bone mass phenotype with notably diminished bone mass. At the cellular level, these alterations were brought about by increased bone resorption by osteoclasts and significantly enhanced bone formation by osteoblasts. Their findings indicate that elevated *Gsα* expression contributes to skeletal abnormalities, wherein bone production occurs at the expense of bone mass (132).

#### 3.5.2 Osteoblast

Joy Y. Wu et al. generated *GsαOsx*KO mice by deleting *Gsα* from cells expressing *Osterix* to study its role in the early osteoblast lineage. Such mice developed severe osteoporosis and fractures at birth and did not survive to weaning period. This phenotype results from impaired bone formation rather than increased bone resorption. Osteogenic differentiation is accelerated, and osteoblasts rapidly differentiate into osteocytes, producing woven bone. Also, the number of committed osteoblast progenitors has reduced in bone marrow stromal cells, and cranial osteocytes in cKO mice and osteoblasts are reduced. Wnt signaling was reduced in the osteoblast lineage, and expression of the Wnt signaling inhibitors sclerostin and dickkopf1 (Dkk1) was significantly increased. Their study suggests that Gsα signaling is essential for early normal bone formation (23).

#### 3.5.3 Osteocyte

Fulzele et al. generated mice lacking Gsα in osteocytes by crossing *Dmp1*-Cre mice with *Gsα* ^flox/flox^ mice. *OCY-Gsα*KO animals have low bone mass and decreased adipose tissue and develop a myeloproliferative phenotype. In these mice, they investigated the impact of Gsα deficiency on the bone marrow microenvironment. The deficiency of Gsα in osteocytes led to an increase in bone marrow, spleen, and peripheral blood, resulting from alterations in the bone marrow microenvironment in mice. This effect was independent of osteoblasts or the Wnt-βcatenin pathway. The authors demonstrated that osteocytes could regulate myelopoiesis through a Gsα-dependent mechanism by secreting multiple factors, including granulocyte colony-stimulating factor (G-CSF) (133).

Subsequently, the authors’ team further studied such mice and found that the cortical bone parameters were significantly reduced in all 7-week-old *Dmp1*KO mice by microCT, with no change in distal femoral trabecular bone volume in females but a significant reduction in distal femoral trabecular bone volume in males. Bone trabeculae were significantly reduced in cKO mice by static histomorphometric analysis. Assays of viable osteoblasts showed a dramatic decrease in the number of osteoblasts per bone perimeter. Dynamic histomorphometric analysis showed a significant decrease in the rate of bone formation and mineral attachment per bone surface in cKO mice. Serum levels of osteocalcin and procollagen type I amino-terminal propeptide PINP were significantly reduced in cKO mice. Differently, the number of osteoclasts per total area was significantly reduced in female mice, whereas no change was observed in the male group. Combined with the authors’ previous study: the deletion of *Gsα* in osteocytes leads to sclerostin expression *in vitro* and *in vivo* (134). Immunohistochemistry also showed a significant increase in sclerostin expression in osteoblasts of *Dmp1*-KO mice. PTH can inhibit sclerostin gene expression through the Gsα signaling pathway (135, 136). Treatment of these animals with sclerostin-neutralizing antibodies rescued the number and size of osteoblasts in the cortex as well as partially rescued the low bone mass phenotype. These data suggest that a deficiency of Gsα in osteoblasts can lead to osteoporosis, in part due to an increase in sclerostin (137).

#### 3.5.4 Mesenchymal cell

Regard et al. used *Prx1*-Cre (138) mice mated with *Gsα* ^flox/flox^ (139) mice to produce *Prx1*-Cre; *Gsα* ^f/-^ and *Prx1*-cre; *Gsα* ^f/f^ mice, which had a similar phenotype: born with webbing between fingers, joint fusion and progressive heterotopic appearance in the soft tissues ossification Extraskeletal mineralization was first detected at days E16.5 and E17.5, accelerated during the perinatal period, and became extensive four days after birth, with mineralization in the interphalangeal area and between the ulna and radius, leading to osseous fusion at P4. At P20, most mutant mice die due to extensive bone and joint fusion and mineralization of tendons. The authors also used *Dermo1*-Cre (mesoderm-derived bone and muscle tissue) or *Ap2*-Cre (more extensive than the former) to remove Gsα, again with a similar phenotype of ectopic ossification, so that Gnas inhibits ectopic ossification in multiple mesenchymal tissues. The authors also performed adenoviral subcutaneous injections of *Ad*-Cre or *Ad*-GFP on 4-week-old *Gnas* ^flox/flox^ mice. They found ectopic osteoblasts and mineralization in dermal and subcutaneous areas six weeks after *Ad*-cre injection, along with extensive ectopic ossification detected. The longer the time, the more severe this induction produced ectopic ossification and invasion of deep muscle tissue. It is shown that loss of Gnas in adult subcutaneous mesenchymal tissue is sufficient to cause ectopic ossification similar to that in POH and AHO. *In vitro*, they isolated bone marrow stromal cells (BMSC) and subcutaneous mesenchymal cells (SMP) from Gnas^flox/flox^ mice, which showed accelerated osteogenic differentiation as well as stronger expression of osteogenic differentiation markers after removal of Gsα with *Ad*-Cre infection. They also improved the phenotype of ectopic mineralization by removing Gli2 (relaying Hh signaling) in mutant mice and found that its removal improved or even rescued ectopic ossification, as did injection of ATO or GANT58, pharmacological inhibitors of Gli, into female mice harboring *Prx1*-Cre; *Gsα* ^f/-^ pups. Inhibition of osteoblast differentiation by ANT-58 was also observed in *Gsα*-deficient BMSCs was observed *in vitro*. In conclusion, their study demonstrated the ability of Gsα to confine bone formation within the skeleton by inhibiting Hh signaling in mesenchymal progenitor cells. In conjunction with their previous studies (22), the researchers determined that Gsα serves as a critical regulator of osteoblast differentiation by maintaining the balance between two essential signaling pathways: Wnt/β-catenin and Hh (24).

Xu et al. removed Gsα from osteochondral progenitor cells by generating *Prx1*-Cre; *Gsα* ^flox/flox^ mice, resulting in accelerated cranial bone formation during craniogenesis and cranial malformation after birth. This is because the loss of Gsα activated the Hh signaling pathway and accelerated osteoblast differentiation and ossification during cranial bone development, but the bone formed was of low quality and low mineral density, which may be due to increased osteoclast differentiation (140).

The premature closure of the cranial suture caused by *GNAS* deficiency was further investigated by Xu et al. The authors found inactivation of the Hh signaling pathway in single-cell sequencing of cranial suture chondrocytes from normal neonatal mice, which, consistent with the previous mention, was associated with the activation of Gsα. The authors deleted *Gsα* in cranial chondroprogenitor cells by crossing *Gsα* ^flox/flox^ with *Prx1* -Cre mice. These mice developed severe cranial and limb bone phenotypes, with most mutant mice dying at P6. *Prx1*-Cre; *Gsα* ^flox/flox^ mice developed premature closure of the cranial suture, similar to the cranial malformation of mutant patients (141). *Prx1*-Cre; *Gnas* ^fl/fl^ mice at postnatal day 14 showed ectopic ossification in the sagittal suture, posterior fontanel, lambdoid suture, and mastoid fontanel. Subsequent histological analysis revealed that the earliest cranial suture phenotype could be observed in E16.5 sections. At P0, the mineralization of cranial cartilage in mice occupied half of the cartilage area, and the mutant cells were hypertrophic and mineralized compared with chondrocytes in normal controls. Next, the authors mated R26RtdTom reporter mice with *Prx1*-Cre; *Gsα* ^flox/flox^ mice. Immunostaining revealed increased expression of *ColX, Osx*, and *OPN* in the mutant chondrocytes, while the mutant chondrocytes did not express osteogenic markers. This suggests that the deletion of *Gsα* within chondrocytes allows them to transform into an osteoblast lineage through hypertrophy. They also used *Sox9*-CreER and *Osx*-Cre mice to study the function of Gsα in chondrocytes and the effect of mutated osteoblasts on chondrocytes, respectively. *Sox9*-CreER; *Gnas* ^fl/fl^ mice reproduced ectopic mineralization in mastoid fontanel after being induced at P6. No chondrogenic mineralization or premature fusion of the cranial suture and fontanel was found in the cranial vault of *Osx*-Cre; *Gnas* ^fl/fl^ mice at P0 compared to control *Osx*-Cre mice; therefore, the authors concluded that premature closure of the cranial suture was the result of ectopic ossification of cranial suture mesenchymal cells and chondrocytes. Next, the authors crossed mice with the Ptch1LacZ allele with *Prx1*-Cre; *Gsα* ^flox/flox^ mice. By X-gal staining, t they found that the cranial suture and fontanelle of the mutant mice had substantial activation of Hh signaling. Also, coronal sections of mammillary fontanelles from *Prx1*-Cre; Gnas^fl/fl^; Ptch1LacZ mice had large numbers of LacZ^+^ chondrocytes, while controls had no LacZ^+^ chondrocytes. These data suggest that the regulation of chondrocyte fate by Gsα is associated with the activation of Hh signaling. Reduction of Gli transcription activity by crossing with a loss-of-function Gli2 allele or injecting GLI1/2 antagonist hindered the progression of cartilage HO in neonatal stage mice (142). This implies that reducing Hh signaling could be an intervention to suppress the cranial deformity caused by *Gnas* deficiency (140).

### 3.6 *XLαs* knockout mouse model

*Gnasxl* ^m+/p-^mice (C57BL/6J genetic background) were generated by disrupting the first exon of *XLαs* on the paternal allele, thus resulting in the global ablation of this protein XLαs. Antonius Plagge et al. initially generated and analyzed the phenotype of *Gnasxl* ^m+/p-^ mice, which exhibited the following characteristics: 1) On the first day of life, *Gnasxl* ^m+/p-^ pups could be identified by their narrow, lean body shape, weak and inactive nature, and limited suckling; 2) growth retardation and high postnatal lethality; 3) impaired suckling activity; 4) significantly reduced blood glucose levels and lower glucagon concentrations compared to wild-type pups, indicating a potential endocrine disorder; 5) diminished interscapular fat pads and lipid depletion in inguinal white adipose tissue; and 6) substantially higher cAMP levels per mg of tissue in BAT of *Gnasxl* ^m+/p-^ pups compared to wild-type pups, while cAMP levels in inguinal white adipose tissue remained unchanged. These findings suggest that XLαs regulate several key postnatal physiological adaptations, including lactation, glycemic control, and energy homeostasis. The increased cAMP levels in BAT of *Gnasxl* ^m+/p^ *^-^* mutant mice and the comparison of their phenotype to Gnas mutants imply that XLαs can antagonize the Gsα-dependent signaling pathway (3). Due to the high mortality rate of these mice after birth, their team further used CD1 genetic background *Gnasxl* ^m+/p-^ mice to try to study the metabolic phenotype of mutant mice after adulthood. The fat content and fat accumulation in the adipose tissue of *Gnasxl* ^m+/p-^ mice were reduced, and the food intake and metabolic rate increased, which may be due to the increased activity of the sympathetic nervous system rather than the autonomous effect of adipocytes, as XLαs is not expressed in the adipose tissue of adult mice. At the same time, the urine norepinephrine level of *Gnasxl* ^m+/p-^ mice increased, but the metabolic responsiveness to β3-adrenergic agonists did not increase. *Gnasxl* ^m+/p-^ mice showed hypolipidemia, increased glucose tolerance, and insulin sensitivity. The authors propose that XLαs is a negative regulatory factor of sympathetic nerve activity in mice (110).

Eaton et al.conducted the first study on the role of XLαs in bone metabolism. They generated two new mutant mice strains (Ex1A-T-CON and Ex1A-T). Paternal inheritance of Ex1A-T led to *Gsα* imprinting deficiency and *XLαs* expression deficiency. In adult mice, paternal inheritance of Ex1A-T resulted in increased metabolic rates, decreased fat mass, adiposity, and bone mineral density, which could be attributed to the absence of XLαs. The authors suggest that XLαs is involved in mediating the metabolism of bone and adipose cells (7).

He et al. delved into the GPCR signaling pathway and the impact of XLαs on PTH. They found that on the second day after birth, *Gnasxl* ^m+/p^ *^-^* mice exhibited hyperphosphatemia, hypocalcemia, and increased serum PTH and 1,25-dihydroxy vitamin D concentrations. At the same time, in *Gnasxl* ^m+/p-^ mice, the ability of PTH to reduce serum phosphate levels was impaired, the abundance of the sodium-phosphate cotransporter Npt2a in the renal brush border membrane decreased, and the excretion of PTH-induced cAMP in urine moderately increased. Furthermore, they observed that both basal and PTH-stimulated IP3 production was suppressed in the proximal renal tubules of *Gnasxl ^m+/p-^* mice; IP3 is a second messenger generated through the Gαq11 signaling pathway. Additional analyses indicated that XLαs enhances Gq/11 signaling, mediating the renal effects of PTH during early postnatal development (27). Then, they discussed the dysregulated actions of fibroblast growth factor 23 (FGF23), which contributes to various inherited diseases and mortality in kidney failure patients. The article examines the molecular mechanisms and signaling pathways governing FGF23 production and highlights the role of XLαs in FGF23 synthesis through a G protein-coupled IP3/PKC pathway. The article also notes that elevated levels of PKC activity in skeletal tissue are found in X-linked hypophosphatemia (XLH) models, while XLαs ablation reduces the FGF23 and phosphate phenotype in these mice (29).

### 3.7 *Nesp55* KO mouse model

Contrary to *XLαs*, *NESP55* is a maternally specific transcript. Plagge et al. generated mice with a *Nesp55*-specific knockout. These *Nesp55* knockout mice developed normally but exhibited abnormal reactions to novel environments in behavioral analyses. These findings suggest that maternally expressed *Nesp55* is crucial in regulating exploratory behavior. Dent et al.expanded on these findings. They use *Nesp* ^m-/p+^ mouse models. It was demonstrated that the absence of Nesp55 led to impulsive choices, which can be assessed through delay discounting tasks. *Nesp* ^m-/p+^ mice were less willing to wait for a delayed, larger reward and instead were more inclined to choose an immediate, smaller reward (143).

Bastepe et al. previously identified two nearly identical microdeletions in the *GNAS* gene, spanning from the NESP55 exon to the antisense exon 3 (delNESP55/delAS3-4). Their research in mice demonstrated that when the region equivalent to delNESP55/delAS3-4 is deleted from the paternal allele (termed DeltaNesp55(p)), the resulting animals remain healthy with no alterations in Gnas methylation patterns. On the other hand, mice with the deletion on the maternal allele (designated as DeltaNesp55(m)) exhibited a complete loss of maternal Gnas methylation imprints. This led to an upregulation of 1A transcription and a decrease in Gsα mRNA levels in the kidney, which consequently resulted in hypocalcemia, hyperphosphatemia, and secondary hyperparathyroidism (85, 144).

### 3.8 *1A* mouse model

Williamson et al. utilized mice lacking exon *1A* (corresponding to *A/B* in humans). These mice exhibited normal methylation in the maternal *1A* differentially methylated region, deregulated paternal *Gsα* expression in brown adipose tissue, and attenuated PTH resistance in *Oed-Sml* mice. These findings support the conclusion that mouse *1A* and human *A/B* transcripts possess a silencing function independent of epigenetic *GNAS* changes, which reduces the biological activity of Gsα (6, 115).

### 3.9 FD-related mouse model

As previously mentioned, the cause of FD is a postzygotic mutation in the Gnas gene. Currently, there are several disease models based on the primary R201 mutation as well as transgenic mouse models.

Saggio et al. generated different mouse strains, EF1α-*Gsα* ^R201C^ and PGK-*Gsα* ^R201C^, with two different backgrounds (FVB and C57/Bl6) and two different constitutive promoters (the human elongation factor 1α (EF1α) and the human phosphoglycerate kinase (PGK). They found that FD lesions in mice replicated the human phenotype and were independent of the genetic background and the type of constitutive promoter used. Mutations in *Gsα* were shown to cause FD, and this is the first mouse model of FD. Notably, this model showed germline transmission, i.e., the mutant transgene could be passed on to offspring, unlike the generally accepted notion that FD in humans is not heritable due to the embryonic lethal consequence of constitutive Gsα activity in all cells. Furthermore, due to the germline inheritance, this mouse model is not predicted to have mosaicism, unlike FD (145).

Zhao et al. generated a conditionally inducible FD mouse model by specifically expressing *Gsα* ^R201C^ in skeletal stem cells, and following induction, both embryonic and adult mice rapidly developed FD bone lesions (146).

Khan et al. created a Knockin mouse model, *Gnas* ^f(201H)^, and studied the expression of the *Gsα* mutation in early osteochondroprogenitor cells, osteoblasts, and bone marrow stromal cells by mating with *Prx1-cre* mice and *Sox9-cre* mice, respectively, mice that recapitulate the human FD signature. The FD mutation in Gsα leads to the upregulation of Wnt/β-catenin signaling in bone and bone marrow stromal cells (BMSCs). Reducing this signaling can rescue the FD phenotype (47).

Xu et al. also employed *Gnas* ^f(R201H)/+^ mice in their research on FD and POH. The activation of Gsα results in FD, which presents itself as craniofacial osteomalacia. Their study demonstrated that Gsα governs intramembranous ossification by modulating Hh and Wnt/β-catenin signaling pathways. Furthermore, small molecule inhibitors targeting Hh and Wnt signaling can effectively alleviate the cranial phenotype in mice caused by loss-of-function or gain-of-function mutations in *Gnas*, respectively (20).

Karaca et al. generated mice with a conditionally active *Gsα* mutant, which was dependent on Cre recombinase (c*Gsα*R201H mice). Subsequently, they crossed these mice with *Prx1*-cre mice. The resulting double mutant progeny displayed shorter limbs, hypertrophy of the growth plate, a narrower chondrocyte zone, and delayed development of secondary ossification centers (46).

## 4 Discussion

The *GNAS* locus, encompassing *Gsα*, *XLαs*, and other products, exerts profound influence over a variety of physiological processes through regulating multitude signaling pathways. Among these, the regulation on bone metabolism is particularly noteworthy. Dysregulation of the *GNAS* gene can lead to various skeletal disorders, highlighting the critical role these gene products play in preserving bone homeostasis and function. Gsα, in particular, stands out as a key orchestrator in regulating osteoblast proliferation through cAMP-mediated signaling. Furthermore, it controls calcium and phosphorus levels through its action on renal tubules. Additionally, Gsα’s interaction with Wnt and Hh signaling pathways further amplifies its multifaceted regulatory capacity in bone metabolism and other physiological processes. Further investigation into additional signaling pathways is necessary to expand our comprehension of bone metabolism regulation.

XLαs, closely associated with Gsα, also contributes significantly in this process. Research into gene models associated with *Gsα* and *XLαs* has yielded a wide spectrum of phenotypes, spanning both endocrine function and bone metabolism. Continued exploration of these signaling pathways and gene models will unveil new insights into the complex regulatory mechanisms governing bone health and development. The study of Gnas-related signaling pathways, diseases, and mouse models is pivotal in advancing our understanding of *GNAS*-related disorders, and consequently facilitate the development of more precise treatments.

This comprehensive review systematically revisits the structure, signaling pathways, bone-related disorders, and associated mouse models of the Gnas locus. As the future of research in this field unfolds, several critical areas of focus have emerged:

1) Elucidating the interactions between Gsα, osteoblasts, osteoclasts, and bone microenvironment: Investigating the intricate mechanisms by which Gsα influences the interplay between osteoblasts, osteoclasts, and the bone microenvironment holds the promise of unveiling novel therapeutic strategies for addressing Gnas-related skeletal disorders.
2) Leveraging recently developed approaches: Single-cell and spatial multi-omics offer the potential to study the fundamental mechanisms of Gnas-related genes at a single-cell resolution. The adoption of these advanced techniques may facilitate the discovery of new insights and therapeutic targets for diseases associated with the Gnas locus.
3) Expanding *in vitro* and *in vivo* research on *XLαs*: The study of XLαs gene function, including the exploration of conditional knockout mouse models, can deepen our comprehension of XLαs’s role in Gnas locus-associated bone diseases. This expanded research horizon could potential avenues for the development of innovative treatment approaches.

In summary, a thorough exploration of these research directions can pave the way for improving our understanding of the molecular mechanisms underlying *Gnas* locus-related skeletal disorders, thereby facilitating advancements in diagnosis, treatment, and disease management.

## Author contributions

QH: Writing – original draft, Writing – review & editing. WY: Writing – original draft, Writing – review & editing. YZ: Writing – original draft, Writing – review & editing. NZ: Writing – review & editing. KW: Writing – review & editing. RZ: Writing – review & editing. ZC: Writing – review & editing.
